# MicroRNAs regulatory networks governing the epigenetic landscape of MEN1 gastro‐entero‐pancreatic neuroendocrine tumor: A case report

**DOI:** 10.1002/ctm2.351

**Published:** 2021-04-06

**Authors:** Ettore Luzi, Luca Pandolfini, Simone Ciuffi, Francesca Marini, Federico Cremisi, Gabriella Nesi, Maria Luisa Brandi

**Affiliations:** ^1^ Department of Experimental and Clinical Biomedical Sciences University of Florence Florence Italy; ^2^ Istituto Italiano di Tecnologia (IIT) Genova Italy; ^3^ Scuola Normale Superiore di Pisa Pisa Italy; ^4^ Division of Pathological Anatomy, Department of Experimental and Clinical Medicine University of Florence Florence Italy

Dear Editor,

PanNENs are histopathologically classified as well‐differentiated pancreatic neuroendocrine tumors (panNETs) or poorly differentiated pancreatic neuroendocrine carcinomas (panNECs) according to the 2010 World Health Organization (WHO) classification system. Pancreatic neuroendocrine tumors (pNETs) occur in 60–70% of multiple endocrine neoplasia (MEN1) patients; about 30% of pNETs undergo a malignant progression, manifesting local or distant metastases, which are one of the main causes of death in MEN1 patients.^1^, ^2^ Their molecular characteristics are still undefined. Moreover, despite the common genetic basis, their clinical phenotype is highly variable among patients, even in the presence of the same mutation, suggesting a possible role of other cofactors and/or epigenetic mechanisms in each individual tumorigenesis process.^3^, ^4^, ^5^ We analyzed, through next‐generation sequencing (NGS), the specific miRNA expression signatures of normal pancreas, gastrinoma, and neuroendocrine pancreatic tumor in a MEN1 patient, whose histopathological examination (via duodenopancreatectomy biopsies) revealed diffuse microadenomatosis with eight macrotumors (>0.5 cm) in the pancreas and four gastrin‐secreting tumors (three in the duodenum and one in the gallbladder) (Figure S1). NGS analysis of normal pancreas and MEN1 tumor samples identified the presence of tumor‐subtype‐specific miRNAs (see Figures 1 and 2). These data open to the possibility of using them for distinguishing different MEN1 gastro‐entero‐pancreatic neuroendocrine tumors (GEP‐NETs). Principal component analysis (PCA) analysis allowed us to confirm that our normalized miRNA expression profiles are able to resolve samples according to their tissue. In particular, while biopsy replicates displayed a low amount of variance (Figure 1A and B), the first two principal components captured 87% of the overall variance and separated the control pancreas from MEN1 pNETs and gastrinomas (Figure 1B). To delve more deeply into these groups, we then proceeded to perform a robust statistical characterization of both subtype‐specific and pan‐cancer miRNAs. We first performed a group‐wise study, in order to extract the miRNAs specifically up‐/down‐regulated in either pNETs or gastrinomas when compared to control pancreas (“pNET vs. CP” and “Gas vs. CP,” respectively). miRNAs with an adjusted *p*‐value < 0.01 were considered differentially expressed (DE) between pairs of conditions (Tables 2 and 3), and are shown in Figure 1C–F. UpSet plot in Figure 2A shows the degree of intersection between up‐ (UP) and downregulated (DOWN) miRNAs in “pNET vs. CP” and “Gas vs. CP” contrasts. Our data demonstrate that most of the miRNAs analyzed are homogeneously split into pNET‐specific, Gas‐specific, and Common classes, while only <4% of them have a divergent behavior. The normalized expression values of miRNAs belonging to the four classes in panel A are shown as heatmaps (Figure 2B–E) and may contain a useful panel of subtype‐specific biomarkers. We then focused on the class of miRNAs DE between MEN1 gastro‐entero‐pancreatic cancer tissues (pNETs and gastrinomas) and control pancreas. miRNAs with an adjusted *p*‐value < 0.01 were considered DE between conditions (Table 4). We observed 17 downregulated and 26 upregulated miRNAs, whose log2 FoldChanges versus average expressions and *volcano plots* are shown in Figure 3A. These data were also shown in a heatmap containing the normalized expression value of differentially regulated miRNAs (Figure 3B). These miRNAs represent a miRNA signature specific for MEN1 gastro‐entero‐pancreatic tumors.

**FIGURE 1:**
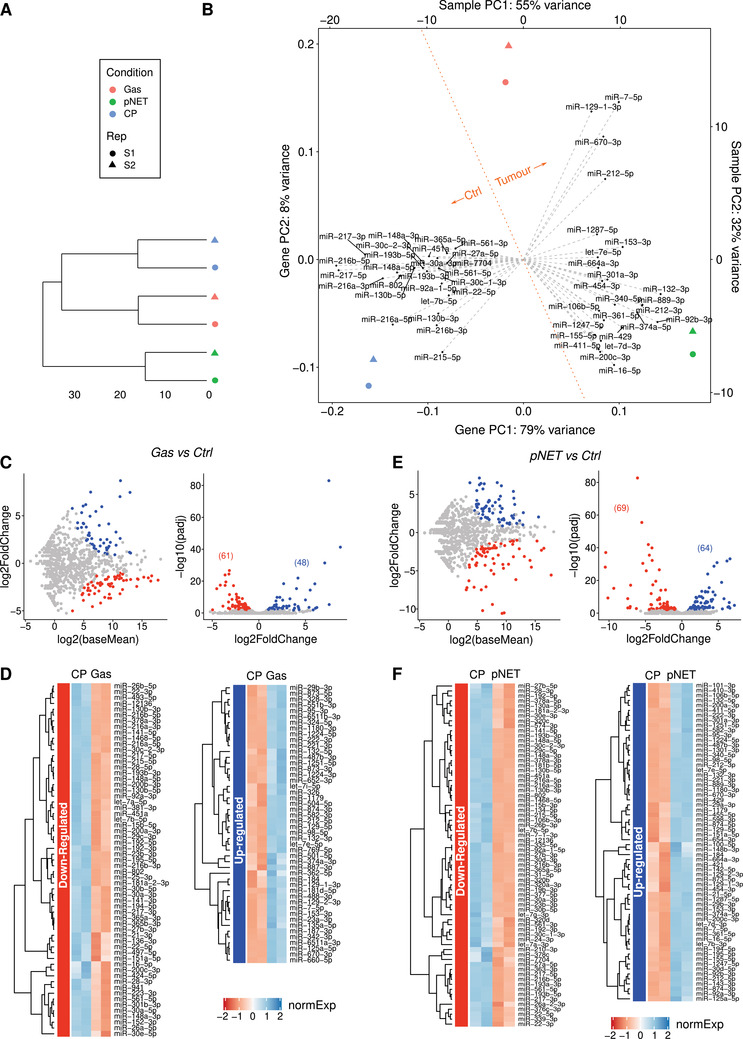
Analysis of miRNA profiles in control and tumor MEN1 samples by NGS. (A) Dendrogram showing the hierarchical clustering of tissue samples, performed according to Euclidean distance and complete linkage. (B) Principal component analysis of miRNA expression across the samples. Gray dashed vectors projecting from the origin reveal how each miRNA contributes to the first two principal components. Each axis shows the percentage of global variance explained by the relative component. Orange dotted line demarcates control (CP) and tumor (pNET and Gas) samples. The description of sample origin for both panels is shown in the legend box. CP, control pancreas; Gas, gastrinoma; pNET, pancreatic neuroendocrine tumor; Rep, replicate; PC1/2, principal component 1/2. (C) miRNA regulation between MEN1 gastrinoma and normal pancreas Left: Logarithmic scatter plot showing miRNA FoldChange as a function of average expression (DESeq2 baseMean value). miRNAs significantly up‐ (UP) or downregulated (DOWN) are shown as blue and red dots, respectively (adjusted *p* value < 0.01). Right: Volcano plot showing the significance of miRNA differential regulation as a function of log2 FoldChange. Between parentheses the number of differential miRNAs are indicated. miRNAs significantly up‐ (UP) or downregulated (DOWN) are shown as blue and red dots, respectively (adjusted *p* value < 0.01). (D) Heatmap representing the normalized expression values (*z*‐scores) of miRNAs differentially regulated between gastrinoma (Gas) and normal pancreas (CP), as shown in panels A and B. Table rows are sorted according to hierarchical clustering, as shown by the left dendrogram. DE, differential expression; n.s., not statistically significant; normExp, z‐scores of normalized expression. (E) miRNA regulation between MEN1 pNET (pancreatic neuroendocrine tumor) and normal pancreas (see description in panel C). (F) Heatmap representing the normalized expression values (*z*‐scores) of miRNAs differentially regulated between pNET (pancreatic neuroendocrine tumor) and normal pancreas (CP; see description in panel F)

**FIGURE 2:**
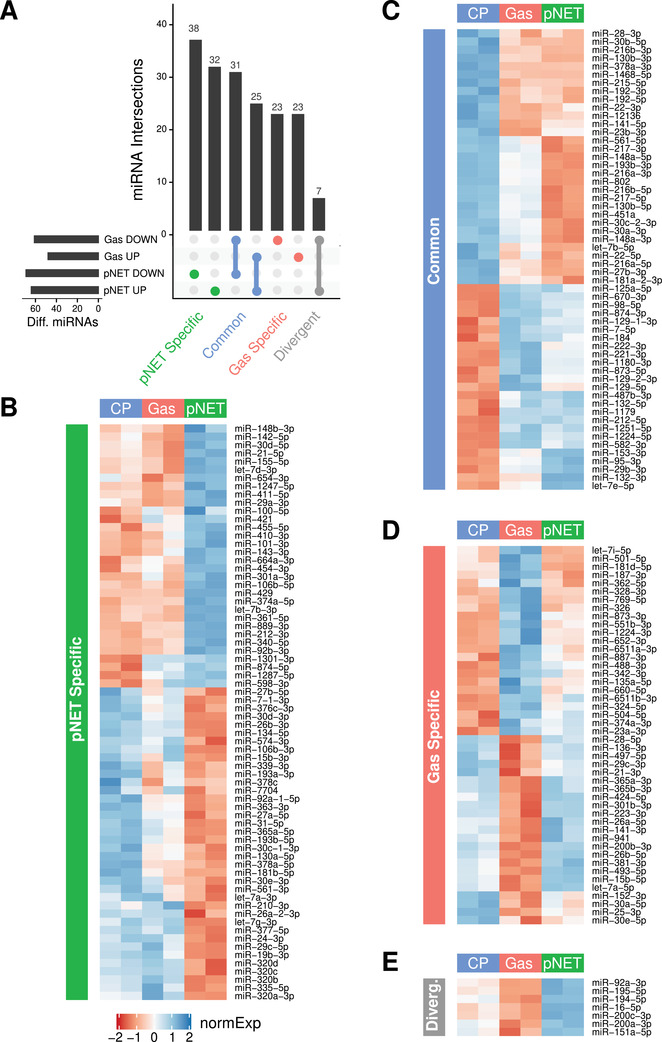
miRNA signatures in MEN1 gastro‐entero‐pancreatic tumor. (A) UpSet‐plot showing the degree of intersection of up‐ (UP) and downregulated (DOWN) miRNAs between “pNET versus CP” and “Gas versus CP” contrasts (adjusted *p* value < 0.01). Most of the miRNAs taken into account are fairly homogeneously split into the pNET‐specific, Gas‐specific, and Common classes, while <4% of them has a divergent behavior (i.e., upregulated in insulinoma and downregulated in gastrinoma). (B–E) Heatmaps representing the normalized expression values (*z*‐scores) of miRNAs belonging to the four classes depicted in panel (A). Table rows are sorted according hierarchical clustering, as shown by the left dendrogram. CP, control pancreas; Gas, gastrinoma; pNET, pancreatic neuroendocrine tumor; normExp: *z*‐scores of normalized expression

**FIGURE 3:**
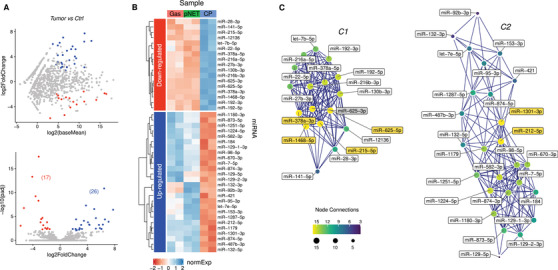
miRNA regulatory network in MEN1 gastro‐entero‐pancreatic tumorigenesis. (A) miRNA regulation between tumor MEN1 (gastrinoma + insulinoma) and normal pancreas. Top: Logarithmic scatter plot showing miRNA FoldChange as a function of average expression (DESeq2 baseMean value). miRNAs significantly up‐ (UP) or downregulated (DOWN) are shown as blue and red dots, respectively (adjusted *p* value < 0.01). Bottom: Volcano plot showing the significance of miRNA differential regulation as a function of log2 FoldChange. Between parentheses the number of differential miRNAs are indicated. miRNAs significantly up‐ (UP) or downregulated (DOWN) are shown as blue and red dots, respectively (adjusted *p* value < 0.01). (B) Heatmap representing the normalized expression values (*z*‐scores) of miRNAs differentially regulated between tumors (gastrinoma + pNET) and normal MEN1 pancreas, as shown in panels (A) and (B). Table rows are sorted according to hierarchical clustering, as shown by the left dendrogram. DE, differential expression; n.s., not statistically significant; CP, control pancreas; Gas, gastrinoma; pNET, pancreatic neuroendocrine tumor; normExp: *z*‐scores of normalized expression. (C) Network of miRNAs differentially regulated between normal and tumoral pancreas, with a correlation ≥0.9. Network nodes are color coded and sized according to their number of connections with adjacent neighbors in order to highlight hubs. miRNA network topology results to be split in two distinct and fairly independent cliques, corresponding to two clusters of anti‐correlated miRNAs

Then, we applied a correlation network model to identify co‐expressed miRNAs and study the impact of their regulation in the context of the network. The reconstructed network originated from MEN1 control and MEN1 tumor gastro‐entero‐pancreatic samples, and focused on DE miRNAs. The topology of this network evidenced two independent cliques (Figure 3C) corresponding to two clusters of anti‐correlated miRNAs, and highlighted some hubs of marked connectivity. We hypothesized that hub miRNAs may act as proxies to help explaining the regulatory function played by the broader regulatory network. Therefore, we examined hub miRNAs from the two different cliques in a deeper analysis.

First, to confirm deep sequencing results, we used qRT‐PCR to assess the expression of seven hub miRNAs from the two different cliques (C1 clicque: miRNA‐378‐3p; miRNA‐1468‐5p; miR‐625‐5p; miR‐625‐3p; miR‐215‐5p. C2 clicque: miR‐1301‐3p; miR‐212‐5p). Importantly, the trend of the alteration in the expression was generally concordant (six out of seven) between the sequencing data and qRT‐PCR (Figure 4A). Moreover, for most of these miRNAs, qRT‐PCR results showed a good agreement with the expression levels found in the BON1 cell line (pancreatic carcinoid tumor), commonly used as a reference model for NETs.

**FIGURE 4:**
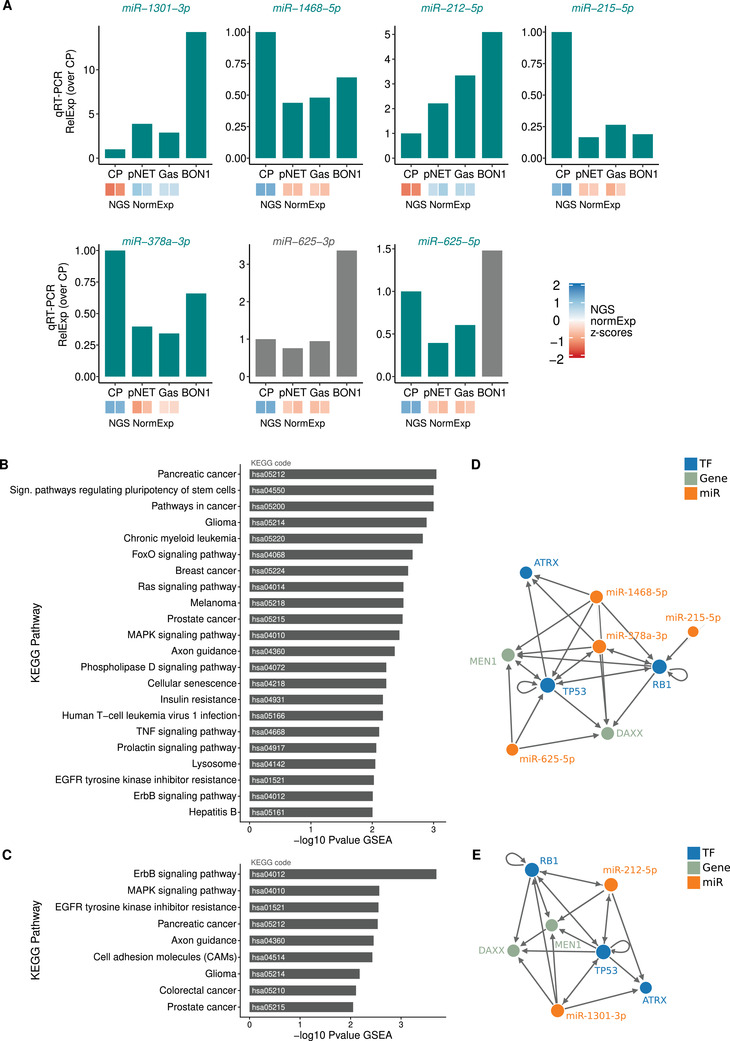
Exploration of miRNA regulatory networks. (A) Validation of NGS‐miRNA differential expression levels by real‐time quantitative RT‐PCR. In cyan are depicted miRNAs showing concordant expression between NGS and qRT‐PCR. Inset boxes show normalized NGS expression values (*z*‐scores). (B and C) Gene‐set Enrichment Analysis (GSEA) of the genes predicted to be targets of the hubs in miRNA regulatory network cliques C1 and C2, respectively (miRNAs marked by a yellow label in Figure 3C) as evaluated by miRWalk 3.0 platform. Only KEGG pathways with an enrichment *p* value < 0.01 are shown. (D and E) Feed‐forward loops (FFL) reconstructed analyzing the interaction between miRNA hubs (as in Figure 3C) and genes involved in MEN1 gastro‐entero‐pancreatic tumor via the FFL tool web server (http://bioinfo.life.hust.edu.cn/FFLtool/)

Next, we checked whether genes predicted to be targets of these hub miRNAs shared a common function. GSEA (Gene‐set Enrichment Analysis) revealed that predicted gene targets (according to miRWalk 3.0) were associated to specific KEGG pathways (Figure 4B and C and Table 5) related to cancer establishment and signaling: noteworthy, the list of the top hits includes the “pancreatic cancer” pathway.

Exerting their role as key determinants of gene expression, transcription factors and miRNAs are able to co‐regulate the expression of targets in the form of feed‐forward loops (FFLs) and feedback loops: feed‐forward loop analysis evidenced interactions between hub miRNAs and the principal genes involved in the MEN1 gastro‐entero‐pancreatic neoplasia, as transcription factors (TF) and/or other mRNAs (Figure 4D and E).^6^

The analysis showed miRNAs “hubs” organized in FFLs with chromatin‐remodeling genes (MEN1, ATRX, DAXX) involved in familial and sporadic cancer, as well as with classical oncogenes (RB, TP53) normally mutated in neuroendocrine carcinomas (NEC) (Figure 4D and E). Therefore, it is tempting to suggest that these epigenetic bistable molecular regulatory circuits^7^, ^8^ could influence the transition from normal neuroendocrine cells to either well‐differentiated GEP‐NET cancer or to a poorly differentiated NEC.

In summary, by investigating global miRNA expression signatures in matched samples of normal pancreas, pNET, and gastrinoma from a MEN1 clinical case, here we highlighted new specific miRNA signatures and confirmed previously selected microRNAs involved in MEN1 gastro‐entero‐pancreatic tumors. These miRNAs have been shown to be organized in two gene regulatory networks with common and distinct functional attributes. While this work clearly represents a starting point, and investigation of a greater number of cases will be required to generalize the results here presented, these data make an interesting exploratory contribution to the knowledge of the mechanisms governing MEN1 tumorigenesis.

## ETHICS APPROVAL AND CONSENT TO PARTICIPATE

The tissue samples for the study were obtained from a female donor undergoing duodenopancreatic surgery in 2002. Samples were collected by the surgeon after verbal informed consent from the patient, who fully understood that the samples were to be analyzed anonymously for future research on MEN1 syndrome not necessarily related to her specific clinical case. All genetic and molecular analyses for this study were carried out anonymously. Researchers had only access to clinical data of the donor and pathologic characteristics of the samples.

## DATA AND MATERIALS AVAILABILITY

Tables containing raw and processed data from the bioinformatic pipeline (including miRNA counts and complete secondary analysis results) can be accessed at the following address: http://doi.org/10.17632/wfm74g24d7.1.

## CONFLICT OF INTEREST

The authors declare no conflict of interest.

## Supporting information

Supporting Information

Supporting Information
