# Supplementary material for: MicroRNAs regulatory networks governing the epigenetic landscape of MEN1 gastro‐entero‐pancreatic neuroendocrine tumor: A case report
Source: Clin Transl Med. 2021 Apr 6;11(4):e351. doi: 10.1002/ctm2.351 (PMC8023566; doi:10.1002/ctm2.351)
Supplement: Supplementary file 1 — Supporting Information [file CTM2-11-e351-s001.doc]

****Supplementary Material****

****Materials and** Methods**

Samples

The tissue samples for the study were obtained from a female donor undergoing duodenopancreatic surgery in 2002. Samples were collected by the surgeon after verbal informed consent from the patient, who fully understood that the samples were to be analyzed anonymously for future research on MEN1 syndrome not necessarily related to her specific clinical case. All genetic and molecular analyses for this study were carried out anonymously. Researchers had only access to clinical data of the donor and pathologic characteristics of the samples.

At the age of 47, the patient was admitted to the Regional Center for Hereditary Endocrine Tumors at the Azienda Ospedaliero-Universitaria Careggi, Florence, with recurrent renal colic since the age of 40, biochemical signs of hypercalcemic primary hyperparathyroidism and duodenal ulcer. The clinical diagnosis of MEN1 syndrome was genetically confirmed by a positive sequencing analysis of the coding region and splicing sites of the *MEN1* gene, revealing the presence of a germinal heterozygote inactivating mutation in exon 6 (c.fs843_844delGA).

The patient underwent total parathyroidectomy with forearm auto-transplantation, partial thyroidectomy and total thymectomy at the age of 52, duodenopancreatectomy at 54 and ablation of a non-functioning adenoma of the anterior pituitary at 58.

Immunohistochemistry

The duodenopancreatectomy specimen was fixed in 10% neutral buffered formalin before being processed in paraffin. Immunohistochemical studies were performed using the streptavidin-biotin-peroxidase method (UltraVision kit; Lab Vision, Fremont, CA) with diaminobenzidine as the chromogen and hematoxylin as the nuclear counterstain. A panel of antibodies against the following antigens was used: chromogranin A (CgA) (clone LK2H10; BioGenex, San Ramon, CA; predilute), synaptophysin (polyclonal; Cell Marque, Hot Springs, AR; predilute), neuron-specific enolase (NSE) (clone E27; Cell Marque; predilute), somatostatin (polyclonal; DAKO, Carpinteria, CA; dilution 1:4), insulin (polyclonal; DAKO; predilute), glucagon (polyclonal; DAKO; dilution 1:4), gastrin (polyclonal; BioGenex; dilution 1:150), pancreatic polypeptide (PP) (polyclonal; DAKO; dilution 1:1000), and Ki-67 (clone 30.9; Ventana, Tucson, AZ; predilute). Positive immunohistochemical controls included normal human tissues to which the appropriate antisera were added. Negative controls were performed by substituting the primary antibody with a nonimmune serum at the same concentration.

RNA extraction

Fresh frozen specimens of one duodenal gastrinoma (Gas), one pancreatic neuroendocrine tumor (pNET), and healthy pancreas tissue (CP), were retrieved from surgery biopsies and stored for biological analyses.

Total RNA was extracted from these three samples using Trizol (Invitrogen, Waltham, MA, USA).

miRNA sequencing

Sequencing procedure was carried out using Illumina Sequencing by Synthesis technology^1^. 1 μg of total RNA was used for library preparation (Illumina, TruSeq Small RNA Sample Prep Kit) following the manufacturer’s description. Libraries were sequenced on a miSeq (Illumina) running in 50 bp single-read mode using sequencing chemistry v3, and demultiplexed in FASTQ format using CASAVA v.1.8 (Illumina). After demultiplexing, each library produced between 0.8M and 2M reads.

qRT-PCR

Quantitative real-time RT-PCR was carried out as it follows. Each RNA sample was analyzed in triplicate (including the normalization control U6). Ten µg of total RNA from each sample were DNAse-treated with a DNA-free kit (Ambion, Applied Biosystems, Foster City, CA USA). Two µl of DNA-free RNA were reverse transcribed by using miScript reverse transcription Kit (Qiagen, Hilden, Germany) and amplified using miScript SYBR Green PCR Kit and specific microRNAs and U6 miScript Primer assay (Qiagen). RNA expression was quantified with MX3000P multiplex quantitative PCR instrument (Stratagene, La Jolla, CA, USA), using MXPro software, following a three-step protocol: one cycle at 95° for 15 min and 40 amplification cycles (94°C for 15 sec, 1 min at 55°C and 70°C for 30 sec). Sample fluorescence was detected during the annealing step. Fluorescence data were collected continuously to obtain the dissociation curve. Fluorescence was plotted versus the threshold cycle (Ct) based on baseline-corrected, reference dye-normalized fluorescence (dRn) to obtain the standard curve and to measure the initial mRNA quantity. Gene expression was normalized to U6 RNA. miRNA expression level was calculated using delta-cycle threshold (ΔCt), which is the Ct of the miRNA of interest subtracted by the Ct value of the endogenous control. Fold difference between samples was determined by the formula: fold change = 2-ΔΔct

Primary bioinformatic analysis

Library adaptors were trimmed with Trimmomatic^2^ and reads were mapped to the human genome (GRCh38/hg38) with STAR^3^ using parameters from the ENCODE guidelines for miRNAs: “*--alignEndsType EndToEnd --outFilterMismatchNmax 1 --outFilterMultimapScoreRange 0 --outFilterMultimapNmax10-outFilterScoreMinOverLread 0 --outFilterMatchNminOverLread 0 --outFilterMatchNmin 16 --alignSJDBoverhangMin 1000 --alignIntronMax 1*”^4^. Mapping reads were annotated according to miRbase22.1^5^ and summarized using featureCounts^6^. Raw read counts are contained in Tab. 1.

Secondary bioinformatic analyses

Secondary bioinformatic analyses were conducted on the R environment for statistical computing and graphics (https://www. R-project.org/) employing the package collection tidyverse^7^ . Normalization and differential expression analysis were performed by Relative Log Expression and negative binomial Wald test, respectively, with package DESeq2^8^. n=2 replicates were analyzed per tissue type. miRNAs with an adjusted p-value < 0.01 were considered differentially expressed between conditions.

miRNA expression heatmaps were plotted using tidyHeatmap^9^, applying row-wise z-score scaling and row sorting according to hierarchical clustering.

miRNA network was obtained filtering miRNAs differentially expressed between tumor (pNET+Gas) and control pancreas (CP), and visualized using package ggnetwork^10^ .

The edges of the network represented miRNA pairs with Pearson correlation >= 0.9.

The degree of intersection of up- and down-regulated miRNAs between pNET *versus* CP and Gas *versus* CP contrasts was displayed *via* an UpSet plot generated by package UpSetR^11^. Gene Set Enrichment Analysis (GSEA) on genes predicted to be targets of the miRNA network hubs was performed using miRWalk 3.0 web server^12^ , implementing the novel comprehensive prediction software TarPmiR^13^ and calculating the statistical significance of enrichment by Fisher exact test.

The reconstruction of Feed-Forward loops was performed *via* the “FFLtool web server”^14^.

Tables containing raw and processed data from the bioinformatic pipeline

(including miRNA counts and complete secondary analysis results) can be accessed at the following address:

1. Bowman S, Simon M, Deaton A, Tolstorukov M, Borowsky M, Kingston R.Multiplexed (2013) Illumina Sequencing Libraries From Picogram Quantities of DNA BMC Genomics 4:466.

2. Bolger, A.M., Lohse, M., and Usadel, B.(2014) Trimmomatic: a flexible trimmer for Illumina sequence data. Bioinformatics 30:2114–2120.

3. Dobin, A., Davis, C. A., Schlesinger, F., Drenkow, J., Zaleski, C., Jha, S., Batut, P., Chaisson, M., and Gingeras, T. R. (2013) STAR: ultrafast universal RNA-seq aligner. Bioinformatics 29:15–21.

4. Davis, C. A., Hitz, B. C., Sloan, C. A., Chan, E. T., Davidson, J. M., Gabdank, I., Hilton, J. A., Jain, K., Baymuradov, U. K., Narayanan, A. K et al. (2018) The Encyclopedia of DNA elements (ENCODE): data portal update. Nucleic acids research 46: D794–D801.

5. Kozomara, A., and Griffiths-Jones, S. (2014) miRBase: annotating high confidence microRNAs using deep sequencing data. Nucleic Acids Res 42:D68–D73.

6. Liao, Y., Smyth, G.K., and Shi, W. (2014;) featureCounts: an efficient general purpose program for assigning sequence reads to genomic features. Bioinformatics 30:923–930

7. Wickham, H. Welcome to the Tidyverse. (2019 ) Journal of Open Source Software 4:1686.

8. Love MI, Huber W, and Anders S. ( 2014) Moderated estimation of fold change and dispersion for RNA-seq data with DESeq2. Genome Biology 15:550.

9.MangiolaS.(2020)TidyHeatmap:ATidyImplementationofHeatmap https://github.com/stemangiola/tidyHeatmap.

10. Briatte F.(2020) ggnetwork: Geometries to Plot Networks with 'ggplot2'.

<https://CRAN.R-project.org/package=ggnetwork>

11. Gehlenborg N.(2019) UpSetR: A More Scalable Alternative to Venn and Euler Diagrams for Visualizing Intersecting Sets. https://CRAN.R-project.org/package=UpSetR

12. Sticht C, De La Torre C, Parveen A, Gretz N (2018).miRWalk: An online resource for prediction of microRNA binding sites. PLoS One. 18:13 :e0206239.

13. Ding J, Li X, Hu H. (2016)TarPmiR: a new approach for microRNA target site prediction. Bioinformatics.32 :2768-75

14. Xie GY, Xia M, Miao YR, Luo M, Zhang Q, Guo AY. (2020)FFLtool: A Web Server for Transcription Factor and miRNA Feed Forward Loop Analysis in Human. Bioinformatics.

*36:2605-2607.*
