# Supplementary figures and images for: MicroRNAs regulatory networks governing the epigenetic landscape of MEN1 gastro‐entero‐pancreatic neuroendocrine tumor: A case report
Source: Clin Transl Med. 2021 Apr 6;11(4):e351. doi: 10.1002/ctm2.351 (PMC8023566; doi:10.1002/ctm2.351)

**Figure S1**

**A**

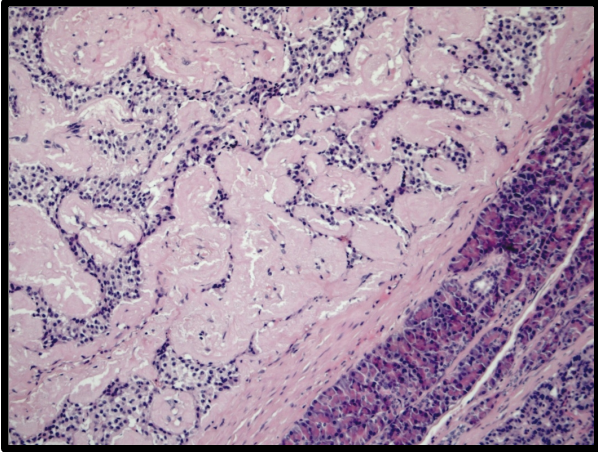

H&E

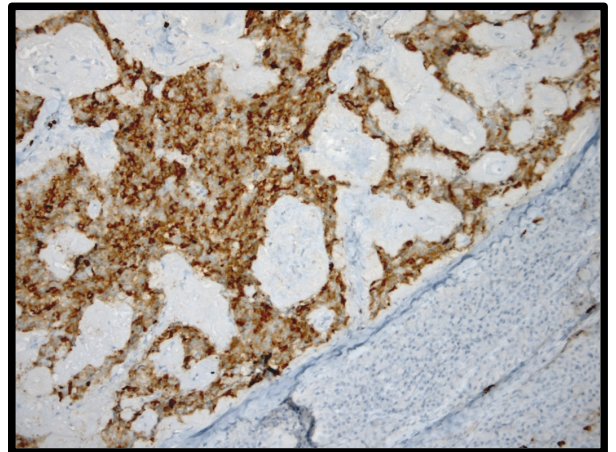

CgA

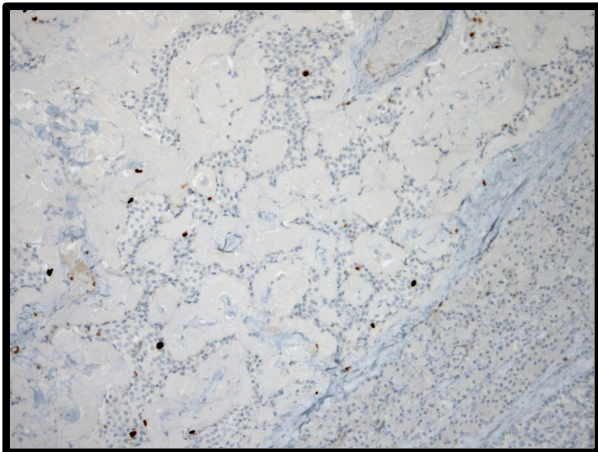

Ki-67

**B**

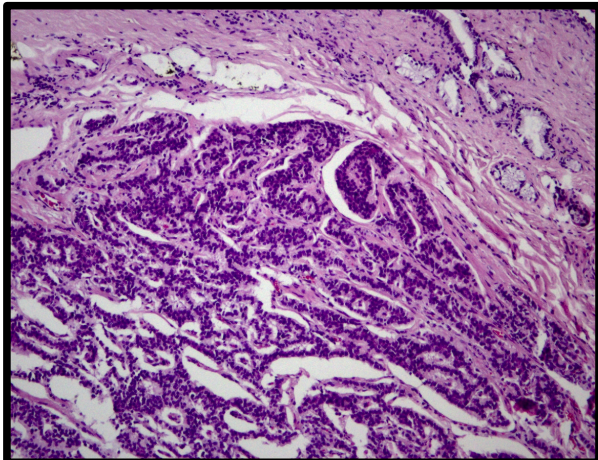

H&E

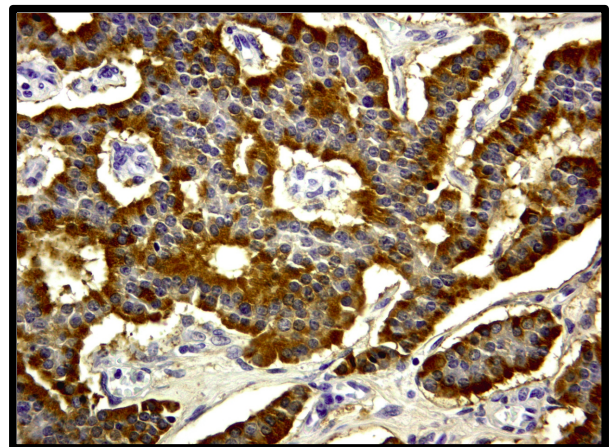

GAST

Supplement: Supplementary file 2 — Supporting Information [file CTM2-11-e351-s002.pdf]
